# A systematic florula of a disturbed urban habitat: Pavements of Sheffield, England

**DOI:** 10.3897/BDJ.4.e10658

**Published:** 2016-10-28

**Authors:** Oliver L. Pescott

**Affiliations:** ‡ Centre for Ecology and Hydrology, Wallingford, United Kingdom

**Keywords:** urban, vascular plants, systematic survey, randomised survey, Sheffield, England, urban ecology, pavement, sidewalk, urban flora, monitoring, baseline study, disturbance

## Abstract

**Background:**

Human settlements are of increasing interest to ecologists, a fact demonstrated by the recent cluster of book-length treatments of the topic (Forman 2008, McDonnell et al. 2009, Gaston 2010, Niemelä et al. 2011, Wilson 2011, Forman 2014). The natural world as a fascinating feature of towns and cities has a much longer history (e.g. Fitter 1945), and has also played a strong part in local biological conservation in some countries over the late 20th Century (Goode 2014​). Despite much existing information on urban plant and animal communities resulting from these trends, very little, easily accessible, systematic data on urban biodiversity is currently available.

**New information:**

Few systematic, randomised surveys at fine spatial grain exist for urban habitats, and even fewer of these surveys are in the public domain. This study was designed as a systematic florula (i.e. a small flora) of a relatively discrete urban habitat in order to provide a baseline that would enable robust insights into future environmental change. In addition, the dataset is likely to be useful for comparative studies of plant traits, particularly those of highly disturbed habitats (Williams et al. 2009​). The survey is an occupancy study of the vascular plants of pavements (i.e. sidewalks) within 16 500 x 500 m (0.25 km^2^) urban grid cells, stratified by quadrant at the scale of the focal city (Sheffield, England) in order to provide more even coverage. The final dataset comprises 862 records of 183 taxa.

## Introduction

Williams et al. (2009) argue that a focus on the floras of urban environments is justified for several reasons: cities are increasingly the main point of contact between humans and the natural world; the inhabitants of cities depend on vegetation to provide numerous ecosystem services, including cultural ones; and urban areas contribute to the conservation of species. The conceptual framework of Williams et al. (2009) details four areas where research on urban floras would benefit from comparative studies; such studies are best enabled by easily accessible and well-documented data.

A considerable amount of information on urban floras already exists, as shown in Britain and Ireland by the number of published Floras of towns and cities (e.g. Shaw 1988, Futter and Raynes 1989, Beesley and Wilde 1997, Dickson et al. 2000, Whild et al. 2011) or of regions which include large conurbations (Burton 1983, Trueman et al. 2013); this is also true on the European continent (e.g. Brodtbeck et al. 1997, Brodtbeck et al. 1999, Landolt 2001, Purro and Kozlowski 2003​), and no doubt elsewhere (see the city floras analysed by Duncan et al. 2011). In Britain at least, these publications have often collated species occurrence data from largely volunteer biological recorders (Pocock et al. 2015), and as such they are typically a mix of so-called 'opportunistic' data, and data that are the result of more structured recording protocols—'checklist' recording of grid cells for example (Pescott et al. 2015). Whilst species occurrence data collected by such projects have often contributed to larger datasets, such as the *New Atlas of the British and Irish Flora* (Preston et al. 2002), there are very few instances of systematic recording schemes of urban environments, with the structure of the recorded data kept intact, being made available outside of regional or national collations.

Although the author is aware of well-documented studies of particular urban habitats (see the bibliography of Shaw 1988 for example), including streets (Chater et al. 2000) and walls (Shimwell 2009), these would require redigitisation efforts from printed lists in order to mobilise collected data for use in comparative ecological studies. Other datasets, apparently in more user-friendly forms, do not appear to be in the public domain (e.g. Kent et al. 1999, Thompson and McCarthy 2008). It is hoped that the data presented here are therefore useful to other ecologists engaged in studies of urban plants.

## General description

### Purpose

To record the vascular flora of a disturbed habitat, in an urban setting, within a systematic, randomised framework.

### Additional information

The resulting species records presented in this data paper have also been shared with the Sorby Natural History Society (www.sorby.org.uk), and the Botanical Society of Britain and Ireland (BSBI) (www.bsbi.org) as 1 km^2^ occupancy data. Through inclusion in the database of the BSBI, these species occurrence records will ultimately form part of a BSBI dataset on the UK National Biodiversity Network (www.nbn.org.uk), which itself is a node of GBIF (www.gbif.org). Note, however, that the species occurrence data available through this route will not be attached to survey metadata essential for their interpretation and use as an individual systematically recorded dataset (i.e. disaggregation would be difficult in the absence of prior knowledge of the protocol of the project), hence its presentation in full here.

## Sampling methods

### Study extent

-1.54360021785, -1.33914691539, 53.3205532457, 53.4364503199 (Long. min, Long. max, Lat. min, Lat. max). See also (**Suppl. material 1**)

### Sampling description

All 1 km^2^ grid cells of the British National grid (OSGB 1936 EPSG:27700) with at least 25% 'built-up' land cover in the Centre for Ecology & Hydrology Land Cover Map 2000 (Fuller et al. 2002​) in the Sheffield area were divided into four quadrants (north-east; south-east; south-west; north-west) centred on the following point: Lat.: 53.378493; Long.: -1.430239 (British National grid reference: 438000, 387000); Smith (2010)﻿ figures the area considered. Within these quadrants, all urban 1 km^2^ cells were numbered and a pocket calculator random number generator function was used to select five for survey; in the final project, four 1 km^2^ cells in the north-east quadrant of the conurbation, which fell into the neighbouring city of Rotherham, were excluded from further consideration; 16 cells were thus sampled in total.

**Figure 1.**
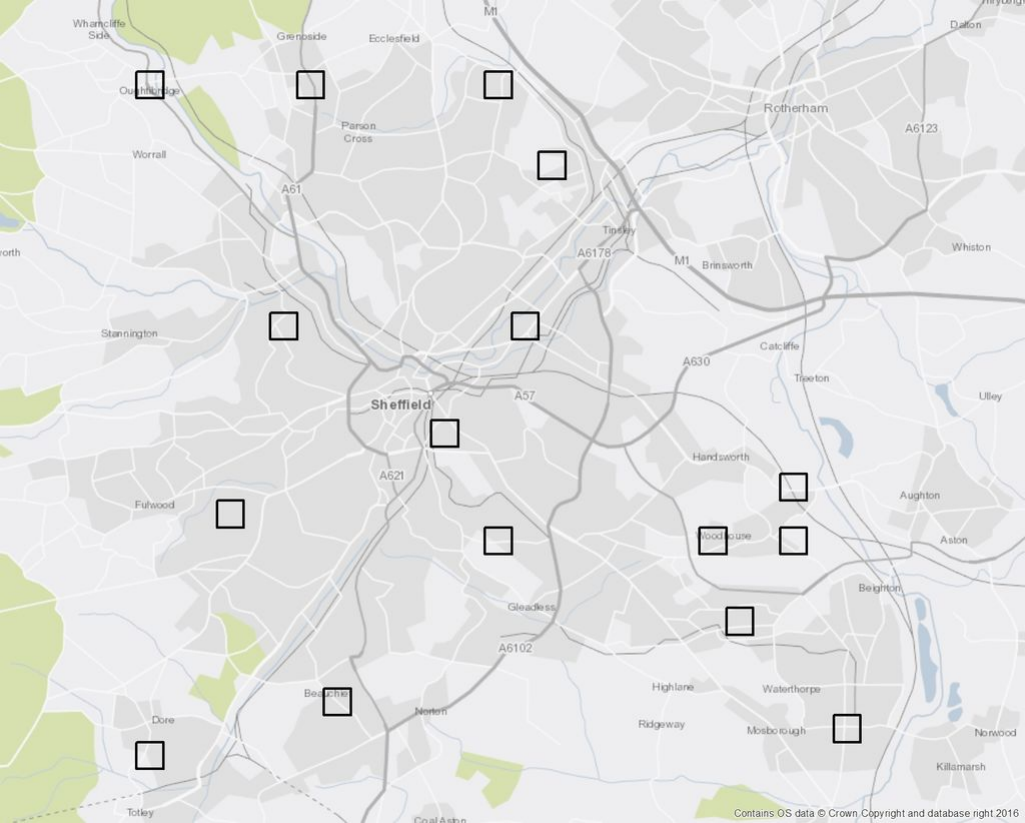
The 16 500 x 500 m sub-cells sampled in Sheffield during this urban recording project. The shapefile is available in (Suppl. material 1).

Within a selected 1 km^2^ urban cell, all four 500 x 500 m (0.25 km^2^) sub-cells with at least 50% built-up area were numbered, with a single sub-cell being selected for survey using the random number method described above. In the field, the 16 selected 500 x 500 m sub-cells (Fig. 1) were surveyed for 1.5 h or until every publicly accessible street had been walked on both sides, whichever was the longer. Plants were only recorded if they occurred in the pavement (sidewalk) habitat; this included plants which were rooted at the edges of the paved area, for example, those immediately abutting walls, grass strips, kerbstones, pavement furniture, utility installations etc. In a small number of cases plant shoots were observed growing on the pavement side of a boundary wall, but which were still rooted in a garden. Such occurrences were recorded, given that they were maintaing a presence in the focal habitat; however, they are marked as 'Garden escapes' in the comments field of the 'Plant occupancy' dataset described below (**Data resources**).

### Quality control

All plants identified in this study were identified by the author. Plant specimens which could not be named in the field were keyed using the current standard Flora for Britain and Ireland (Stace 2010) or a vegetative key (Poland and Clement 2009​). In a very small number of cases where the available material was not sufficient for certain identification, specimens were recorded to genus level (also see **Temporal coverage** below).

## Geographic coverage

### Description

The city of Sheffield, England.

### Coordinates

53.3205532457 and 53.4364503199 Latitude; -1.54360021785 and -1.33914691539 Longitude.

## Taxonomic coverage

### Description

This study includes all vascular plants found growing in target habitat; 183 taxa were recorded in total. See **Sampling methods** above for more information on habitat recording criteria.

## Temporal coverage

**Data range:** 2012 6 23 – 2014 7 11.

### Notes

This survey was intended as a cross-sectional 'snapshot' of the habitat of interest, therefore the surveys were constrained to the period of late June and early July in all three years of the survey. Although this means that a small number of late-flowering species (e.g. *Conyza* spp.) were only identified to genus level (where vegetative identification was assessed to be uncertain), the consistency in survey period should have reduced issues with species detectability due to phenology within this study (multiple repeat visits to sub-cells at different times of year were outside of the resources of this unfunded project). Future re-surveys of this habitat in Sheffield should take this temporal restriction into account, particularly if phenological shifts due to climatic changes have shifted the corresponding period of growth to different calendar dates.

## Usage rights

### Use license

Open Data Commons Attribution License

## Data resources

### Data package title

A systematic florula of a disturbe﻿d urban habitat, Sheffie﻿ld, England﻿: Plant occupancy data

### Resource link

https://catalogue.ceh.ac.uk/documents/705d6e39-9b04-497d-b8e8-4f617f3a6477

### Alternative identifiers

705d6e39-9b04-497d-b8e8-4f617f3a6477

### Number of data sets

1

### Data set 1.

#### Data set name

Plant_﻿occupancy_data﻿

#### Data format

CSV

#### Number of columns

9

#### Character set

UTF-8

#### Download URL

https://catalogue.ceh.ac.uk/download?fileIdentifier=705d6e39-9b04-497d-b8e8-4f617f3a6477

#### Data format version

1.0

#### Description

Vascular plant occupancy data. Taxonomy and nomenclature follow Stace 2010, except for *Sagina apetala* *sensu lato* (= *Sagina apetala* *sensu stricto* and *Sagina filicaulis* of Stace 2010) and *Polygonum aviculare* agg. (= *Polygonum aviculare* *sensu stricto* and *Polygonum arenastrum* of Stace 2010).

**Data set 1.** 

Column label	Column description
recordedName	Speci﻿e﻿s or species aggregate as recorded in the field.
nameAuthor	Authority for name, where appropriate.
date	date (DD/MM/YYYY)
1kmLocation	Lettered British National grid reference for sampled 1 ﻿km square.
1kmQuadrant	Quadrant (i.e. ﻿500 x 500 m ﻿sub-cell) of 1 ﻿km square sampled.
centroidEasting	Easting component of centroid of 500 x 500 m sampled sub-cell.
centroidNorthing	Northing component of centroid of 500 x 500 m sampled sub-cell.
buffer (m)	Distance (in metres) by which centroids should be buffered and squared off in order to recreate the sampled areas. See Suppl. material 1. for an ESRI Shapefile of these areas.
comment	Comments relating to species occurrence observations﻿ made in the field.

## Supplementary Material

Supplementary material 1 Surveyed grid cell data Data type: ESRI Shapefile
Brief description: An ESRI Shapefile (EPSG: 27700) containing spatial polygons delimiting the sampled areas (500 x 500 m sub-cells; Fig. 1) of this study. Attribute table columns (title; contents) are: 1km_square (1 km^2^ cell reference according to the lettered format of the Britian National grid); Quadrant (compass-point quadrant, i.e. 500 x 500 m sub-cell sampled); Location (textual description of geographic location); Ref (reference to this data paper).
File: oo_98431.zip O.L. Pescott
